# Analysis of outer membrane protein from *Orientia tsutsugamushi* for epitope mining

**DOI:** 10.6026/973206300213140

**Published:** 2025-09-30

**Authors:** Jogender Yadav, Sudheer Gupta, Ankur Joshi, Abhijit P. Pakhare, Priyal Gupta, Santenna Chenchula, Shashank Purwar

**Affiliations:** 1 Department of Microbiology, All India Institute of Medical Sciences Bhopal, Madhya Pradesh, India; 2 NGS & Bioinformatics Division, 3B BlackBio DX Ltd., Bhopal, Madhya Pradesh, India; 3 Department of Community and Family Medicine, All India Institute of Medical Sciences Bhopal, Madhya Pradesh, India; 4 Department of Pharmacology, All India Institute of Medical Sciences Bhopal, Madhya Pradesh, India

**Keywords:** *Orientia tsutsugamushi*, Immune epitope, B-cell Epitope, T-cell Epitope, 56 kDa gene

## Abstract

Scrub typhus, caused by *Orientia tsutsugamushi*, is an emerging health threat that can lead to severe complications if untreated. Therefore, it is of interest to analyse 130 sequences of the 56 kDa outer membrane protein, a key immunodominant antigen. SNP profiling identified 300 conserved and 8 highly variable residues. Epitope prediction revealed 73 conserved and 6 variable B cell epitopes, along with 4 conserved and 1 variable CD8^+^ T cell epitopes and 3 conserved and 1 variable CD4^+^ T cell epitopes. Notably, five immunogenic regions were predicted to activate B cells and both CD8^+^ and CD4^+^ T cells simultaneously. Thus, we show valuable insight into immune recognition and evasion strategies of *O. tsutsugamushi*. Subject to experimental validation, the identified epitopes represent promising candidates for rational vaccine development, improved serodiagnostics and novel therapeutic interventions against scrub typhus.

## Background:

Scrub typhus, caused by *Orientia tsutsugamushi* and transmitted by the larval stage of trombiculid mites, is a re-emerging infectious disease predominantly affecting rural regions across the Asia-Pacific. It is a major cause of acute febrile illness (AFI), often presenting with nonspecific symptoms and, in some cases, an eschar. If untreated, it can progress to severe complications, including multi-organ failure, with mortality rates ranging from 7-30% [1, 2]. Globally, approximately one billion people are at risk and an estimated one million cases occur annually [2, 3-4]. Multiple genes in *O. tsutsugamushi* have been investigated for their roles in pathogenicity and immune recognition, including the 22 kDa, 47 kDa, 56 kDa, 58 kDa, 110 kDa and Sca family proteins (ScaA-ScaE). Among these, the 56 kDa outer membrane protein is particularly notable for its high antigenicity and sequence variability [5, 6]. This gene, with an open reading frame of about 1600 bp encoding a 516-541 amino acid protein, is implicated in host cell invasion via fibronectin binding. It also contains four hypervariable regions (I-IV), which significantly contribute to its antigenic diversity [7, 8]. To date, more than 40 genotypes of *O. tsutsugamushi* have been identified based on variation in the 56 kDa gene [9]. Analysing both conserved and variable regions of the 56 kDa gene is crucial in epitope prediction studies for vaccine development, understanding immune response and designing diagnostic tools [5, 6]. Therefore, it is of interest to analyse the outer membrane protein from *Orientia tsutsugamushi* for epitope mining.

## Methodology:

## Sequences retrieval:

A systematic search was conducted in the National Centre for Biotechnology Information (NCBI) [10] database to retrieve 130 full-length amino acid sequences derived from the 56 kDa gene of *Orientia tsutsugamushi*. The search was performed using the keyword *Orientia tsutsugamushi* 56 kDa gene "complete" in FASTA format. These sequences were exclusively sourced from human specimens collected between 1993 and 2024. The Gene Bank sequence AP008981.1 was used as the reference sequence.

## MSA and SNP analysis:

Multiple sequence alignment (MSA) and single-nucleotide polymorphism (SNP) analysis were performed using the resources available at the Bacterial and Viral Bioinformatics Resource Centre (BV-BRC). For aligning the sequences, MAFFT was used as the alignment algorithm [11, 12, 13, 14-15]. All 130 sequences were examined for MSA and SNP analysis to determine the entropy score, which facilitated the identification of genetic variations within the 56 kDa gene of *Orientia tsutsugamushi*. Based on the MSA and SNP analysis, the entropy score was calculated using the SNP analysis tool. The entropy score represents the distance between the amino acid residues in different sets of homologous protein sequences. Based on the range of entropy scores, they were subdivided into bins: 0-50, 51-100, 101-150, 151-200 and above 200. Residues with an entropy score ranging between 0 and 50 were considered conserved, while residues with an entropy score greater than 200 were considered highly variable. On the other hand, the variable category (51-200) was divided into three groups: Category 1 (51-100), Category 2 (101-150) and Category 3 (151-200).

## B-Cell epitope prediction:

B-cell epitope (BCE) prediction was performed using the Immune Epitope Database and Tools (IEDB) [16]. Predictions were generated through four distinct methods available in the IEDB: Parker Hydrophilicity Prediction, Kolaskar & Tingaonkar Antigenicity, Karplus & Schulz Flexibility Prediction and Chou & Fasman Beta-Turn Prediction. The cut-off values were 1.704, 1.031, 0.997 and 0.971, respectively. These cut-off values were the default settings, as they were optimised during the standardisation and validation of the tool. A site was considered a BCE if the score was above the cut-off value. A consensus result was established by considering a region as a B-cell epitope site if it elicited positive predictions from three or more of the aforementioned methods.

## MHC-I epitope prediction:

Using the Immune Epitope Database and Tools (IEDB), MHC class-I epitope prediction was conducted. Predictions were generated through four distinct methods, namely NetMHCpan 4.1 BA [17, 18, 19, 20-21], ANN [22, 23, 24, 25, 26-27], SMM [28] and SMMPMBEC [29], which are available in the IEDB. The predictions were carried out for the 27 alleles recommended by the IEDB [16].

## MHC-II epitope prediction:

The prediction of MHC class-II epitopes was also carried out using the IEDB platform. Four different prediction methods, namely NetMHCIIPan 4.1 BA, NetMHCIIPan 4.3 BA, NetMHCIIPan 4.2 BA [17, 30, 31, 32, 33- 34] and NN-align 2.3 [33, 35], were employed. The prediction was conducted for the 27 alleles suggested by the IEDB. Both MHC class I and MHC class II results were analysed based on the IC50 (half-minimal inhibitory concentration) value. A lower IC50 value indicates stronger binding efficiency of the peptide to the HLA molecule, while a higher IC50 value indicates weaker binding efficiency. According to the method's recommendations for both MHC class I and II peptides, if the IC50 value of any peptide is equal to or less than 50, it is considered a strong binder. Peptides with IC50 values between 51 and 500 are considered intermediate binders, while peptides with IC50 values greater than 500 are considered weak/non-binders [16].

## Results and Discussion:

In this study, the reference sequence of the 56 kDa antigen of *Orientia tsutsugamushi* comprised 516 amino acids. Among these, 300 residues exhibited low entropy scores (<50), indicating high conservation across different strains. In contrast, 8 residues showed high entropy scores (>200), signifying considerable variability. Complete entropy data are presented in Figures 1-3 (see PDF) and Supplementary File 1. Using four different prediction methods, we identified 179 B-cell epitope (BCE) residues that were predicted by at least three methods. Of these, 73 were located in conserved regions, while 6 were within highly variable regions. These findings are illustrated in Figure 4 (see PDF) and detailed in Supplementary File 2. In Figure 4 (see PDF), BCEs in conserved regions are marked in yellow (with green highlighting zero-entropy residues), while red indicates BCEs in variable regions. T-cell epitope prediction was also performed. For MHC class I, 508 peptides were identified across 27 common alleles, with 33 classified as strong binders. Among these, four peptides were located in conserved regions: MLIASAMSA (HLA-A02:03, HLA-A02:06), LIASAMSAL (HLA-A02:03), TLAAGMTIA (HLA-A02:03) and AMSALSLPF (HLA-A32:01, HLA-B15:01). One strong binder, RPRQDLNIL (HLA-B*07:02), was identified in a highly variable region (Table 1 - see PDF and Supplementary File 3). For MHC class II, 502 peptides were predicted, of which 142 were strong binders. Three strong binders were located in conserved regions: MKKIMLIASAMSALS (HLA-DRB108:02), KIMLIASAMSALSLP (HLA-DRB109:01) and LIASAMSALSLPFLA (HLA-DQA105:01/DQB103:01). One peptide, ADGKKHLSLITGIPF (HLA-DRB1*07:01), was found in a highly variable region (Table 2 - see PDF and Supplementary File 4).

We further analysed regions with overlapping B-cell and T-cell epitopes. These combined antigenic sites are promising vaccine candidates as they could stimulate both humoral and cellular immune responses. Five strong MHC class I peptides also overlapped with BCEs and strong MHC class II binders: VLSDKITKI (HLA-A02:03), YTSGKIDGV (HLA-A02:06, HLA-A68:02), KIYSDIRQF (HLA-A32:01), MITGVESTR (HLA-A68:01) and LMASVGVRY (HLA-B15:01) (Table 3 - see PDF). Identifying conserved epitopes is crucial for designing vaccines and diagnostics with broad strain coverage. Conversely, studying variable regions helps us understand immune escape mechanisms that may contribute to disease severity or persistence. The TSA56 protein, a dominant surface antigen constituting ~20% of the bacterial proteome, remains a major focus for vaccine and serological assay development [5, 36]. The 300 conserved residues identified in our study offer a strong foundation for further exploration of immune targets. Meanwhile, the 8 highly variable residues could contribute to strain-specific immune responses or immune evasion strategies. B-cell epitopes play a central role in antibody-mediated immunity, which is vital in the defence against *O. tsutsugamushi* [37, 38]. Previous studies have demonstrated the importance of B-cell responses in acquired immunity [6, 39, 40]. For instance, Namsa *et al.* [6] proposed several B-cell epitopes, including QQQGQVTAQEA, FSKIEEKYSINP, PISIADRDFGIDIPN and PFGGTLAAGMTI. We found conserved residues within these sequences. However, two peptides reported by them-GGNAFANQIQLNF and TDSEGKKHLSLTTG-were located in highly variable regions and were not predicted as B-cell epitopes in our analysis. Similarly, Dolley *et al.* [36] identified a linear B-cell epitope (VGETKADSVGGKDAPI) that binds a Karp strain-specific monoclonal antibody. Our findings showed this region contains variable residues, indicating that some strains may evade recognition by such antibodies. T-cell immunity is also crucial for protection [5, 6, 36, 41, 42-43]. N-Y Ha *et al.* [5] identified two MHC class I peptides-YLRNISAEV and QLYKDLVKL-that triggered robust IFN-γ responses in recovered patients. We found YLRNISAEV to be a strong MHC class II binder, though it was located in a variable region, while QLYKDLVKL was not predicted as a strong binder in our study. Notably, the same study reported MLIASAMSA (HLA-A*0201) as a strong MHC class I binder, which we also identified in a conserved region. Ramaiah *et al.* [8] reported CD4^+^ T-cell epitopes from Indian and South Korean isolates, identifying 68 and 14 epitopes, respectively. We found overlaps with 23 of the 68 Indian epitopes and 13 of the 14 South Korean epitopes, although none were located in conserved regions. To our knowledge, this is the first comprehensive study analyzing variability within the 56 kDa antigen and identifying both conserved and variable regions capable of inducing B-cell, CD4^+^ and CD8^+^ T-cell responses. These epitopes hold promise as vaccine candidates and diagnostic markers.

## Limitations:

This study has some limitations. We focused only on the 56 kDa antigen, rather than the whole proteome. Because entropy scores apply to individual positions, we assigned each BCE residue its own entropy score, but for MHC peptides (9-15 residues), we used the highest entropy value within each peptide. Finally, our predictions covered only 27 common MHC alleles, so some relevant epitopes could have been missed.

## Conclusion:

We identified 6 immunogenic regions in 56 kDa protein that represent potential protective antigens against *O. tsutsugamushi*. We also identified several conserved as well as highly variable immune epitopes which can induce B cell, CD8^+^T cell and CD4^+^ T cells. Subject to experimental validation, these proposed protective antigen epitopes could be valuable for development of vaccine and serology or point of care test against *Orientia tsutsugamushi* infection.
